# Quick high-temperature hydrothermal synthesis of mesoporous materials with 3D cubic structure for the adsorption of lysozyme

**DOI:** 10.1088/1468-6996/16/2/024806

**Published:** 2015-04-28

**Authors:** Geoffrey Lawrence, Arun V Baskar, Mohammed H El-Newehy, Wang Soo Cha, Salem S Al-Deyab, Ajayan Vinu

**Affiliations:** 1 Australian Institute for Bioengineering and Nanotechnology, The University of Queensland, #75 Corner Cooper and College Road, Brisbane 4072, QLD, Australia; 2 Petrochemical Research Chair, Department of Chemistry, College of Science, King Saud University, Riyadh 11451, Saudi Arabia

**Keywords:** mesoporous, nanoporous, adsorption

## Abstract

Three-dimensional cage-like mesoporous FDU-12 materials with large tuneable pore sizes ranging from 9.9 to 15.6 nm were prepared by varying the synthesis temperature from 100 to 200 °C for the aging time of just 2 h using a tri-block copolymer F-127(EO_106_PO_70_EO_106_) as the surfactant and 1,3,5-trimethyl benzene as the swelling agent in an acidic condition. The mesoporous structure and textural features of FDU-12-HX (where H denotes the hydrothermal method and X denotes the synthesis temperature) samples were elucidated and probed using x-ray diffraction, N_2_ adsorption, ^29^Si magic angle spinning nuclear magnetic resonance, scanning electron microscopy and transmission electron microscopy. It has been demonstrated that the aging time can be significantly reduced from 72 to 2 h without affecting the structural order of the FDU-12 materials with a simple adjustment of the synthesis temperature from 100 to 200 °C. Among the materials prepared, the samples prepared at 200 °C had the highest pore volume and the largest pore diameter. Lysozyme adsorption experiments were conducted over FDU-12 samples prepared at different temperatures in order to understand their biomolecule adsorption capacity, where the FDU-12-HX samples displayed high adsorption performance of 29 *μ*mol g^−1^ in spite of shortening the actual synthesis time from 72 to 2 h. Further, the influence of surface area, pore volume and pore diameter on the adsorption capacity of FDU-12-HX samples has been investigated and results are discussed in correlation with the textural parameters of the FDU-12-HX and other mesoporous adsorbents including SBA-15, MCM-41, KIT-5, KIT-6 and CMK-3.

## 1. Introduction

Ever since the discovery of the electron microscope, microscale and nanoscale materials have gained much prominence. Numerous investigations have been reported on controlling their properties by synthesis [1]. Nanoarchitectonics has given rise to several manipulation possibilities right from atoms and molecules to chemical modifications through controlled supra-molecular self-assembly processes to create new functional materials [2, 3]. Both inorganic and organic structures including porous nanomaterials have adopted the concept of ‘nanoarchitectonics’, which helped to develop new nanostructures with improved properties and their relation to the applications of the materials including drug delivery, sensing and cell growth and cell imaging [4, 5]. Mesoporous materials in particular have caught the attention of researchers from various fields and academia and are also presumed to be easy-to-make bulk nanostructures since they are produced remarkably from low cost materials through facile procedures such as mixing, heating, filtration and washing [6]. Mesoporous molecular sieves have excellent textural properties and different functions that make them vital for various applications such as catalysis, adsorption, sensing, and immobilization of biomolecules. In particular, mesoporous materials with three-dimensional (3D) cage-type structures including SBA-6, SBA-16, FDU-1, KIT-5, and FDU-12 are considered more attractive than their uni- and two-dimensional counter parts like MCM-41 and SBA-15 [7–10]. Unlike uni- and two-dimensional mesoporous molecular sieves, the shape and size of the mesopores in a 3D network structure and the connectivity between adjacent pores are considered crucial for applications involving better mass transport and diffusion [11, 12]. Mesoporous silica materials with 3D voids such as MCM-48, SBA-2, SBA-12 and FDU-1 are believed to have more advantages for catalysis and adsorption as their structures are more resistant to pore blocking and offer more adsorption sites and faster diffusion of reactant molecules. One of the important parameters of these materials is the size and shape of the pores which has a direct relation with the specific surface area and pore volume that control the catalytic or adsorption performance [13]. In addition, the spatial distribution of the necks, their sizes and their interconnection with large cavities in porous silica materials are also recognized as the key factors in adsorption studies [14].

The pore diameter of the mesoporous materials can be controlled by either adding swelling agents or using surfactants with different molecular weight or chain length [15, 16]. However, this approach ends up eventually resulting in the loss of structural ordering as it is gradually progressed by instigating pore enlargement as evidenced in the case of MCM-41 and SBA-15 [17, 18]. Zhao and his co-workers adopted a new strategy of combining salt and swelling agents to create a new mesoporous material (FDU-12) with large mesopores, cubic cage-type architecture and Fm3m symmetry [10]. FDU-12 is quite attractive not only because it has large pore entrance sizes [19] but also exhibits high specific surface area, controllable pore cage and entrance dimensions [20–26], and high thermostability [27]. Moreover, it can be easily synthesized from inexpensive reagents which are commercially available. This makes FDU-12 a unique and excellent support for the immobilization of biomolecules [10, 28], catalysis, and sensing applications [29]. Although FDU-12 has excellent structural and textural properties, the time required for its synthesis is long compared to that of its uni- and two-dimensional counterparts such as MCM-41 and SBA-15. The synthesis time can be reduced by simply varying the synthesis temperature. This simple method has gained paramount interest among researchers as it was found to be quite simple and cost-effective. In addition, tuning the dimensions of the pores of various mesoporous siliceous materials and the interstitial voids that link them can be significantly altered with this simple approach [13].

Researchers mostly use synthesis temperatures less than 150 °C for tuning the pore size of the mesoporous materials. This is mainly due to the fact that most of the surfactants decompose at temperatures higher than 130 °C. Zhao and his co-workers also realized this opportunity to tune the textural parameters of FDU-12. However, they used a long reaction time which might destroy the structure of the materials. In this work, we report on the synthesis of large pore FDU-12 materials at different temperatures between 100 and 200 °C. In order to avoid the complete decomposition of the template, the synthesis time has been reduced significantly from 72 to 1 h but at the same time the synthesis temperature has been raised above 150 °C. This simple approach offers a huge reduction in the synthesis time that reduces the timeframe for synthesis of FDU-12 by about 97%, without posing any threats to the structural order but at the same time offering ultra-large cage type pores. We also systematically investigate the possible influence on the structure and textural parameters of FDU-12 samples by shortening the synthesis time and the variation of temperature. The prepared materials have also been used as adsorbents for the adsorption of lysozyme. It has been found that the materials with ultra-large pores prepared at high temperature exhibit a very high adsorption capacity for the lysozyme molecule.

## 2. Experimental section

Pluronic F-127 triblock copolymer (EO_97_PO_69_EO_97_, molecular mass = 12 500) as the structure directing agent and tetraethyl orthosilicate as the silica source were purchased from Sigma–Aldrich. Similarly, sodium bicarbonate, sodium hydroxide and chicken egg white lysozyme (E.C. 3.2.1.17) were purchased from Sigma–Aldrich. All the chemicals were used as received without any further purification.

### 2.1. Synthesis of FDU-12

The steps followed in ultra-fast synthesis of FDU-12 samples with different pore sizes are as follows: 1 g of the triblock copolymer F-127, 1 g of 1,3,5-trimethylbenzene and 1.5 g of KCl were dissolved in 60 ml of 2 M HCl and the mixture was continuously stirred in a closed PPE container at room temperature. About 4.15 g of TEOS was added to the reaction mixture and the stirring was continued at 35 °C. The solution was then transferred into autoclaves and aging was done at different temperatures ranging from 100 to 200 °C for 2 h instead of the conventional duration of 72 h. The white precipitate recovered after filtration was washed with water and dried at 100 °C in a hot air oven prior to calcination. The dried sample was made into a fine powder using a mortar and pestle and then subjected to calcination at 540 °C in air to obtain mesoporous silica FDU-12-HX, where X denotes the synthesis temperature.

### 2.2. Characterization

Structural elucidation of the FDU-12-HX samples prepared at different synthesis temperatures was done using the powder x-ray diffraction (XRD) technique on a Rigaku diffractometer with a Cu K*α* radiation of 0.154 nm. With step time of 1 s and 2*θ* step size of 0.01 the diffractogram was recorded in the range from 0.3 to 10° in the 2*θ* region. Micromeritics ASAP 2420 surface area and porosity analyzer was used to obtain the textural properties of the FDU-12-HX samples and the measurements were done at −196 °C. Degassing was done for all the samples prior to analysis at 250 °C for 4 h. The Brunauer–Emmett–Teller (BET) specific area was obtained from the adsorption branch of the isotherm. The structural morphology of the FDU-12-HX was investigated by using a JEOL JSM 6610 HRSEM operated at an acceleration voltage of 15 kV and a working distance of 8 mm. The internal pore arrangements of FDU-12-HX samples was observed using HRTEM imaging in a JEOL JSM 2100 microscope operated at an acceleration voltage of 200 kV. Samples for HRTEM imaging were prepared by sonication with ethanol for 3 min and deposition over a copper grid. Plausible effect of synthesis temperature on the Si coordination of the FDU-12-HX framework was observed using the ^29^Si magic angle spinning nuclear magnetic resonance (MAS NMR) on a Bruker Avance III 300 MHz NMR spectrometer with a frequency of 59.63 MHz for Si. Samples were rotated in a 4 mm zirconia rotor at a frequency of 7 kHz. With high-power decoupling, the single-pulse experiments were performed using a 4.5 *μ*s pulse with delay time of 100 s. The decoupling scheme used was tppm15. All spectra were referenced against tetramethylsilane (TMS, 0 ppm) and about 800 scans were accumulated.

### 2.3. Lysozyme adsorption

Lysozyme solutions were taken in series with concentration ranging from 0.25 to 4 g l^−1^ for adsorption studies. This was achieved by dissolving different quantities of lysozyme in 25 mM buffer solution using a sodium bicarbonate buffer of pH 11. 20 mg of the adsorbent (FDU-12-HX) was suspended in 4 g of the lysozyme solution with concentration ranging from 0.25 to 4 g l^−1^ in each adsorption experiment. The mixture was consequently shaken continuously in a shaking bath with a speed of 160 shakes min^−1^ at 25 °C until equilibrium was reached at 96 h. The amount of lysozyme adsorbed was studied by subtracting the concentration in the supernatant liquid after adsorption from the amount which was initially present by using optical absorption at 281.5 nm. The instrument was calibrated before each set of measurements, using lysozyme solution buffered at the same pH as the isotherm. To avoid interference from the suspended scattering particles, centrifugation was done prior to the analysis in the UV–vis instrument. The equilibrium time required for maximum adsorption was found to be 96 h and hence it was followed for all the adsorption experiments [30].

## 3. Results and discussion

**Figure 1.**
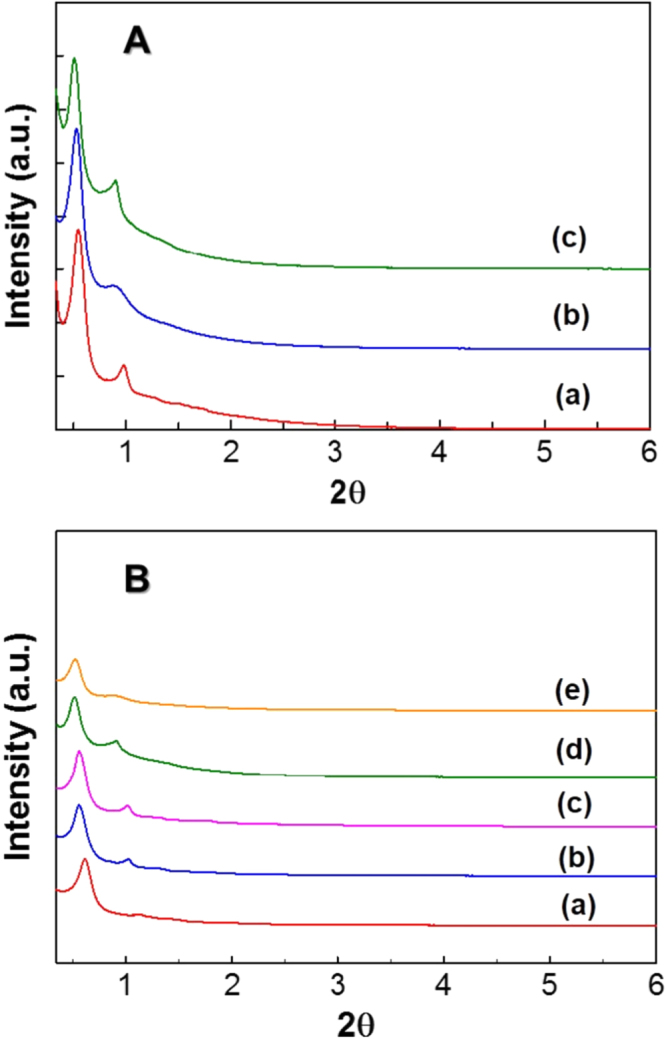
(A) XRD patterns of (A) FDU-12-H200 prepared at the aging durations of (a) 1 h (b) 2 h and (c) 4 h, and (B) FDU-12-H prepared at different temperatures with an aging duration of 2 h. (a) 100 °C, (b) 130 °C, (c) 150 °C, (d) 180 °C, and (e) 200 °C.

Since the synthesis was done at high temperatures for a shorter duration, it is important to check the mesostructure of the prepared samples in order to make sure that the structure of the samples is developed. Initially, the effect of synthesis time affecting the structural and textural properties of FDU-12 samples was studied by preparing FDU-12 samples at a synthesis temperature of 200 °C under different aging periods ranging from 1 to 4 h. Figure 1(A) shows the XRD patterns of FDU-12-H200 samples prepared at different aging periods. It can be seen that irrespective of such rapid aging duration, each of the FDU-12-H200 samples exhibited a characteristic (111) peak followed by reflections corresponding to (220), (311), (331) and (442) lattice planes, which is the characteristic behaviour of the face centred cubic structure [7]. Among the FDU-12-H200 samples prepared at different aging times, the sample prepared with the aging time of 2 h seemed better than those with the aging time of 1 and 4 h as reflected from their respective XRD peak intensities and the well-ordered structure formation. This was due to the fact that the synthesis time of 1 h is not enough for the structure formation, which is due to the poor condensation of silica species, and the synthesis period of 4 h is too long as it destroys the structure at the reaction temperature of 200 °C.

Figure 1(B) shows the XRD patterns of FDU-12-HX samples prepared at different synthesis temperatures ranging from 100 to 200 °C. Irrespective of their synthesis temperatures, all the samples except FDU-12-H100 show highly ordered cubic structure indexed by (111), (220), (311), (331) and (442) lattice planes as shown in figure 1(B), revealing that the structure of the materials is highly developed within a short period of time. FDU-12-H100 exhibits only a sharp peak at low angle with a lower intensity as compared to that of other samples. These results clearly reveal that the synthesis time of 2 h at 100 °C is not enough for the complete development of well-ordered mesostructures whereas the high temperature helps the formation of mesostructures in a shorter duration through extensive silanol group condensation. It should also be noted that the (111) diffraction peak of FDU-12-HX samples is shifted to lower 2*θ* angle with the increase of synthesis temperature, which clearly indicates that the unit cell increases with increasing the temperature. The unit cell, which is calculated from the formula *a*_0_ = *d*√3, increases from 14.5 to 17.3 nm with increasing synthesis temperature from 100 to 200 °C (table 1). Although the aging time is reduced by 36 fold, the 3D mesoporous silica exhibits good structural stability revealing the *h k l* planes of (111) (220) and (311) even at 200 °C. This certainly shows that the templates are quite stable even at such a high temperature although they ought to, at temperatures beyond 150 °C. The probable fact as seen from the literature is purely because of the really short time, which is not enough for the complete decomposition, or the breakdown of surfactant molecules that still support the formation of the mesostructures. In addition, quick condensation of the silica sources at high temperature also helps the formation of mesostructures at relatively shorter synthesis times [31].

**Table 1.** Textural parameters of FDU-12-H prepared at different temperatures with an aging duration of 2 h (*A*_BET_—specific surface area; *V*_p_—specific pore volume, *d*_p_—pore diameter).

Material	*A*_BET_ (m^2^ g^−1^)	*V*_p_ (cm^3^ g^−1^)	*d*_p_ (nm)	*d*_111_ spacing (nm)	Unit cell *a*_o_ (nm)
FDU-12-H100	547.2	0.39	9.9	14.52	25.18
FDU-12-H130	620.2	0.46	11.5	15.64	27.09
FDU-12-H150	947.5	0.73	11.8	15.69	27.18
FDU-12-H180	635.0	0.80	12.2	15.76	27.30
FDU-12-H200	387.0	0.93	15.6	17.33	30.02

**Figure 2.**
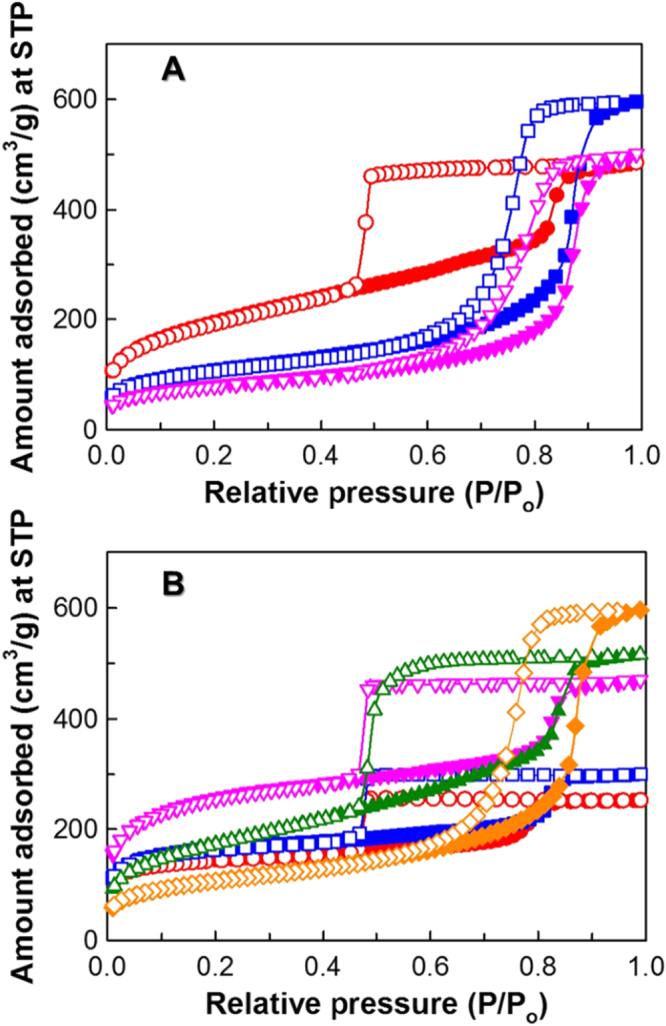
Nitrogen adsorption–desorption isotherms of (A) FDU-12-H200 with different aging durations. (●) 1 h (■) 2 h, and (▼) 4 h and (B) FDU-12-H at different temperatures with an aging duration of 2 h. (●) 100 °C (■) 130 °C (▼) 150 °C (▲) 180 °C and (♦) 200 °C. STP stands for standard temperature and pressure.

**Figure 3.**
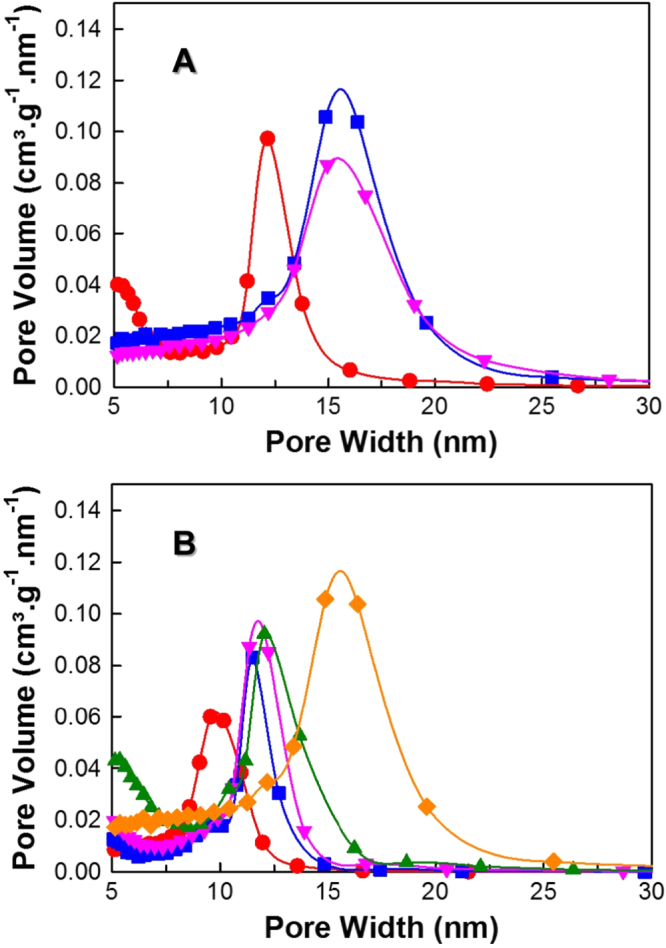
Pore size distribution of (A) FDU-12-H200 prepared with aging durations of (●) 1 h (■) 2 h and (▼) 4 h and (B) FDU-12-H at different temperatures with an aging duration of 2 h (●) 100 °C (■) 130 °C (▼) 150 °C (▲) 180 °C and (♦) 200 °C.

The effect of the synthesis time on the textural parameters of FDU-12-HX was assessed by nitrogen adsorption–desorption measurements. Figure 2(A) shows the N_2_ adsorption–desorption isotherms of FDU-12 synthesized at 200 °C for different synthesis times. The isotherms of all the calcined samples are of type IV isotherm with a pronounced capillary condensation step at a higher relative pressure, revealing the presence of large pore systems. However, the broadness of the hysteresis loop is decreased with increasing synthesis time. The type of hysteresis loop also changes from H2 to H1 upon increasing the synthesis time from 1 to 4 h. These results reveal that the shorter synthesis time favours the cage type pores as it gives a H2 type hysteresis loop whereas enlarged large cage type pores with narrow pore size are obtained when the synthesis time was increased above 1 h. It should also be noted that the total amount of nitrogen adsorbed for the samples prepared with the aging time of 2 h is higher as compared to that of other samples, indicating that the reaction time of 2 h is the best condition to obtain samples with a large pore volume. When the syntheses were performed at a high temperature with the aging time of 1 and 4 h, samples with lower pore volume were obtained. It is also interesting to note that the BET surface area decreased from 677.3 to 290.4 m^2^ g^−1^ with increasing aging time from 1 to 4 h, whereas the pore volume and pore diameters exhibited a maximum capacity, such as 0.93 cm^3^ g^−1^ and 15.6 nm, for the sample with an aging period of 2 h (table 2). It can be seen from the pore size distribution (figure 3(A)) that the peaks are sharper for the sample prepared with the shorter duration, showing better structural order of the mesoporous network. From the nitrogen adsorption and XRD results, it can be concluded that the aging time of 2 h at a temperature of 200 °C is the best condition to obtain a highly ordered FDU-12 sample with larger pore volume and ordered pores.

**Table 2.** Textural parameters of FDU-12-H200 prepared with different aging durations (a) 1 h (b) 2 h, and (c) 4 h.

Aging time (h)	*A*_BET_ (m^2^ g^−1^)	*V*p (cm^3^ g^−1^)	*d*_p_ (nm)
1	677.3	0.75	12.2
2	387.0	0.93	15.6
4	290.4	0.78	15.4

In order to understand the role of synthesis temperature on the structure and pore diameter of FDU-12, the samples prepared at different temperatures starting from 100 to 200 °C at a constant aging time of 2 h were analyzed by nitrogen adsorption–desorption measurements; the results are shown in figure 2(B). All the samples displayed type IV isotherms at all synthesis temperatures with broad hysteresis loops. It should be mentioned that the samples synthesized from 100 to 180 °C showed H2 type hysteresis whereas a H1 hysteresis loop was seen for the silica sample prepared at 200 °C. The shape, type and broadness of the hysteresis loop in the isotherm of the samples reveal that the cage type pores are maintained up to a synthesis temperature of 180 °C. However, when the synthesis temperature is increased to 200 °C, the pores are enlarged and connected, without loss of cage periodicity.

The presence of cage type structures was also confirmed by the XRD results. It is also interesting to note that the capillary condensation step is shifted towards higher relative pressure as the synthesis temperature is increased. When the synthesis temperature of 200 °C was used, the sample with the largest pore diameter and pore volume was obtained. However, the specific surface area of the FDU-12-H200 is much lower than that of other samples. This could be due to the presence of large pores in the sample that significantly decrease the specific surface area. The shift of the capillary condensation step towards the higher relative pressures supplements the XRD patterns, which also show enlargement of the mesopores with the increase of *d* spacing unit cell size with increasing synthesis temperature. The increase of the temperatures also causes the increase of the size of the entrance to these pores, which is seen by the narrowing width of the hysteresis loop for higher temperatures and is probably due to the slight decomposition of F127 into small organic molecules that act as swelling agents to enlarge the pores [31]. It is also surmised that the high temperature increases the volume fraction of the hydrophobic part of the surfactant as a result of the transformation of polyethylene oxide moieties of the surfactants into hydrophobic and the decrease of the interaction of these species with the silica species. These effects also support the enlargement of the pores in the samples prepared at high temperature. From the N_2_ adsorption–desorption isotherms of FDU-12-HX samples, it is seen that with increasing synthesis temperature from 100 to 150 °C, the BET surface area also increased from 547.2 to 947.5 m^2^ g^−1^. However, when the temperature was increased above 150 °C, the BET surface area dropped to 635.0 and 387.0 m^2^ g^−1^ for FDU-12 synthesized at 180 °C and 200 °C, respectively. However, the pore diameter and pore volume of the sample increase at higher synthesis temperature.

The pore size distribution of all the samples (figures 3(A) and (B)) reveal that the pores are highly uniform but are slightly broadened with increasing synthesis temperature. The pore volume of all the samples shows a steady increase from 0.39 to 0.93 cm^3^ g^−1^ and so does the pore diameter with a gradual increase from 9.9 to 15.6 nm with increasing temperature from 100 to 200 °C. The logic behind the expansion of the pores in terms of pore cavity volume and pore entrance is that, with the rise of the synthesis temperature, the pore expansion takes place from the remnants released due to gradual decomposition of the template which enlarges the pores and the total pore volume of the samples [31]. Among all the samples prepared, FDU-12-H200 prepared with a synthesis time of 2 h shows the highest pore volume and the largest pore diameter with well-ordered porous structure. These results reveal that the FDU-12 sample with excellent structure order can be synthesized within 2 h of synthesis time by simply increasing the synthesis temperature to 200 °C. This was simply due to the high energy at high temperature that helps the quick condensation of the silanol group and the mesostructure formation within a short duration. This unique approach of high temperature synthesis with a low synthesis time would significantly reduce the total duration of the synthesis time and cost of the whole production process, which paves the way for easy commercialization.

**Figure 4.**
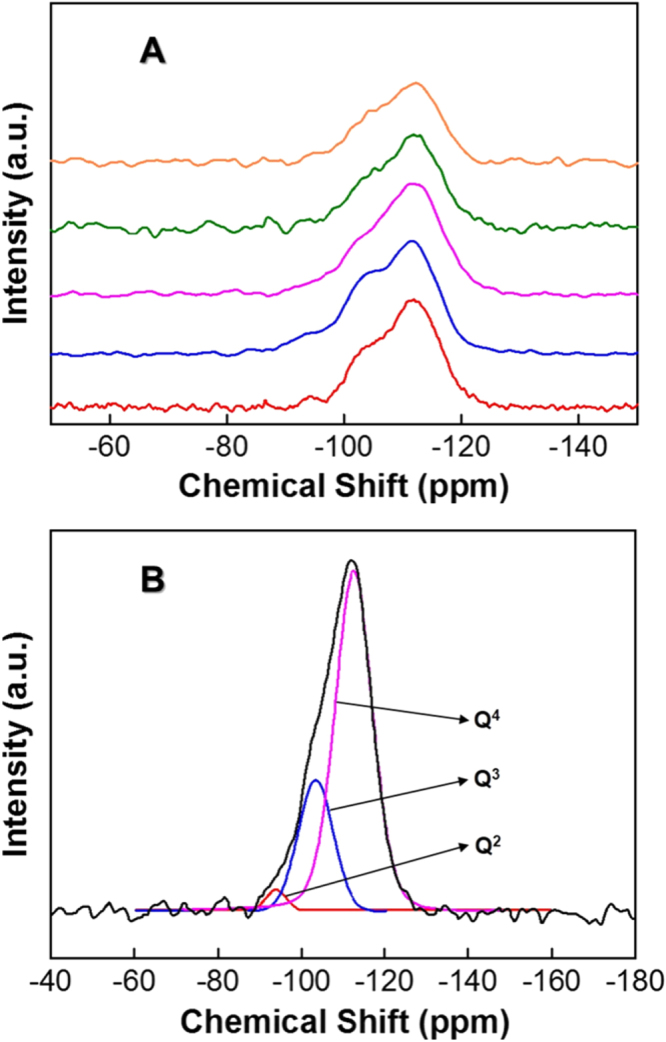
^29^Si MAS NMR spectra of (A) FDU-12-H prepared at different temperatures with an aging duration of 2 h (a) 100 °C (b) 130 °C (c) 150 °C (d) 180 °C, and (e) 200 °C, and (B) deconvoluted ^29^Si MAS NMR spectra of FDU-12-H200.

In order to further understand the silanol group condensation at different synthesis temperatures and the quick formation of mesostructures at high synthesis temperature, the samples were analyzed by the solid state ^29^Si MAS NMR and the data are given in figure 4(A). The spectra were deconvoluted into three well-resolved peaks of chemical shifts around −93, −103 and −112 ppm which corresponds to Q^2^, Q^3^, Q^4^, respectively (figure 4(B)). Q^2^(Si(OSi)_2_(OH)_2_) denotes Si bound to two Si–O moieties and two OH groups whereas Q^3^(Si(OSi)_3_(OH)) represents Si bound to three Si–O moieties and one OH group. On the other hand, Q^4^(Si(OSi)_4_) denotes Si species bound tetragonally to four other Si–O groups [31]. The percentage areas of different Si bonding in the wall structure of the samples prepared at different temperatures are given in table 3. As can be noted in table 3, when the synthesis temperature is increased, the percentage area of the Q^4^ increases with the concomitant decrease of Q^3^ and Q^2^. This confirms the enhancement of the condensation of silanol groups to Si–O–Si when the synthesis temperature of FDU-12 is high. It should also be noted that the decrease in silanol groups in the samples prepared at high temperature offers a high hydrophobic surface which is good for the adsorption of biomolecules [31]. In addition, a high degree of silanol condensation in the wall structure within a short duration of time at high temperature particularly favours other application possibilities for FDU-12 with cage-type pores because these highly cross-linked siloxane moieties offer high hydrothermal stability due to strong Si–O–Si bonding but a lower number of Si–OH groups in the walls.

**Table 3.** The percentage area of different Si species obtained from the ^29^Si MAS NMR spectra of FDU-12-H prepared at different temperatures with an aging duration of 2 h.

Material	Q^4^ (%Area)	Q^3^ (%Area)	Q^2^ (%Area)
FDU-12-H100	61.75	30.82	7.43
FDU-12-H130	67.1	29.46	3.44
FDU-12-H150	70.86	26.50	2.64
FDU-12-H180	73.86	23.91	2.23
FDU-12-H200	75.42	22.94	1.64

**Figure 5.**
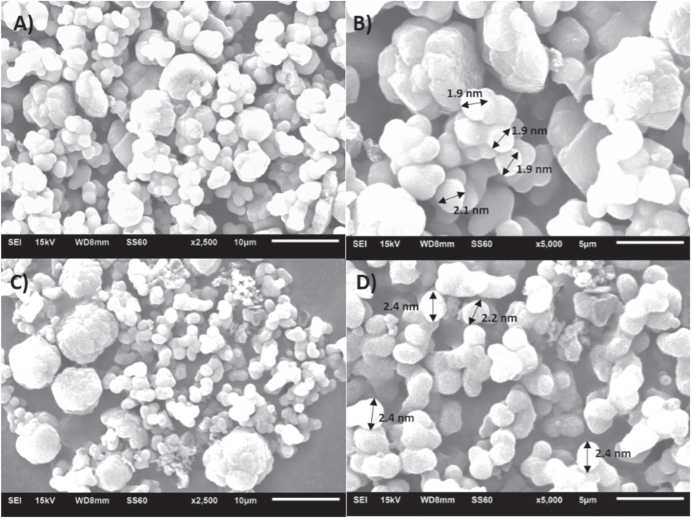
HRSEM images of FDU-12-H prepared at 100 °C (A), (B) and 200 °C (C), (D) for an aging duration of 2 h.

The surface morphology of the calcined FDU-12 samples prepared at different temperatures was obtained using SEM and the images are shown in figure 5. The images obtained show a spherical morphology for the FDU-12 samples indicating the high morphological order of the synthesized materials. From the SEM images, the morphology of the spherical structures seems to exhibit an enlargement in the size of the particles from 1.95 *μ*m for FDU-12-H100 to 2.35 *μ*m for FDU-12-H200 which is in concordance with the insights derived from XRD and BET. The possible reasoning would be that at high reaction temperatures, through liquid crystal assembly, the charged surfactant molecules and the interaction between the silica species are quite notably enhanced. In addition, the enlargement of the surfactant micelles reduces the surface curvature which also results in an increase of the particle size for the sample prepared at high temperature. However, it should be noted that the particles are agglomerated and the spherical shape which is typically obtained for FDU-12 samples is not clear for these samples. This could be due to the short aging time and the high energy at high temperature that favours quick condensation with the neighbouring particles. This would significantly affect the morphological ordering of the samples [31].

**Figure 6.**
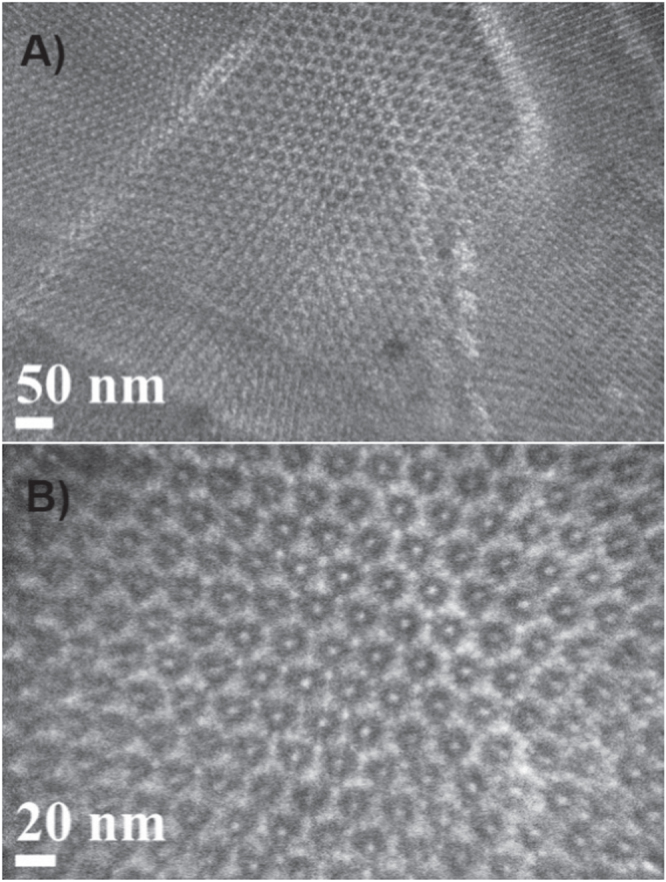
HRTEM images of FDU-12-H200.

The TEM images in figure 6 give a much clearer picture of the FDU-12-H200 synthesized at 200 °C with a greater magnification where we can infer the presence of well defined, uniform cubic structure with Fm3m symmetry with highly ordered array pores over large domains. In FDU-12-H200, the highly ordered pores are arranged in an orderly fashion and connected over significantly large-scale domains. Moreover, due to the short aging duration, even at high temperatures such as 200 °C, the internal porous network remains stable without any detrimental effect. A high magnification of the TEM image also confirms that the mesopores are organized in a 3D network, and the value of the pore diameter is almost similar to that obtained using nitrogen adsorption analysis. All these results reveal that the FDU-12 materials with 3D structure and large pore and pore volumes can be prepared within a short duration by simply increasing the synthesis temperature above 180 °C.

### 3.1. Structural confirmation of the immobilized protein

**Figure 7.**
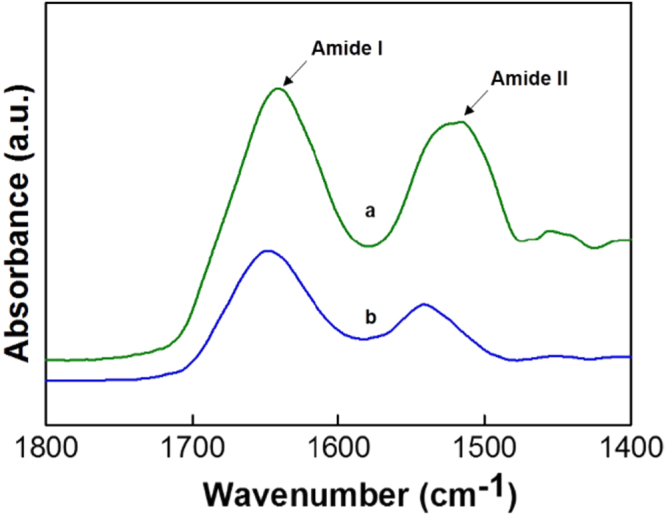
FTIR spectra of pure lysozyme (a) and lysozyme loaded on FDU-12-H200 (b).

The structural stability of the lysozyme immobilized into the pores of FDU12-200H was confirmed through Fourier transform infrared (FTIR) absorption studies. Here, a comparison is drawn between pure lysozyme protein and the lysozyme immobilized into the silica matrix. As can be seen in figure 7(a), the FTIR spectra show distinct amide I and amide II bands, which are characteristic of the structure of the enzyme depicting the confirmation of the *α*-helical and *β*-sheets of the lysozyme. The amide I of the lysozyme, which is observed near 1650 cm^−1^, is attributed to the most sensitive part of the spectral region given by the C=O stretching of the peptide linkages. The amide II found near 1520 cm^−1^ is attributed to the NH bending vibrations of the lysozyme molecule. It is also evident from figure 7(b) that the two amide bands are observed near 1650 and 1550 cm^−1^. It is seen that the intensity ratio of the amide bands in both pure lysozyme and the lysozyme immobilized into the FDU-12 matrix is almost same, which reveals that the lysozyme molecules are quite stable as seen through the secondary structures such as the *α*-helical and *β*-sheets of the immobilized enzyme [32].

### 3.2. Lysozyme adsorption

**Figure 8.**
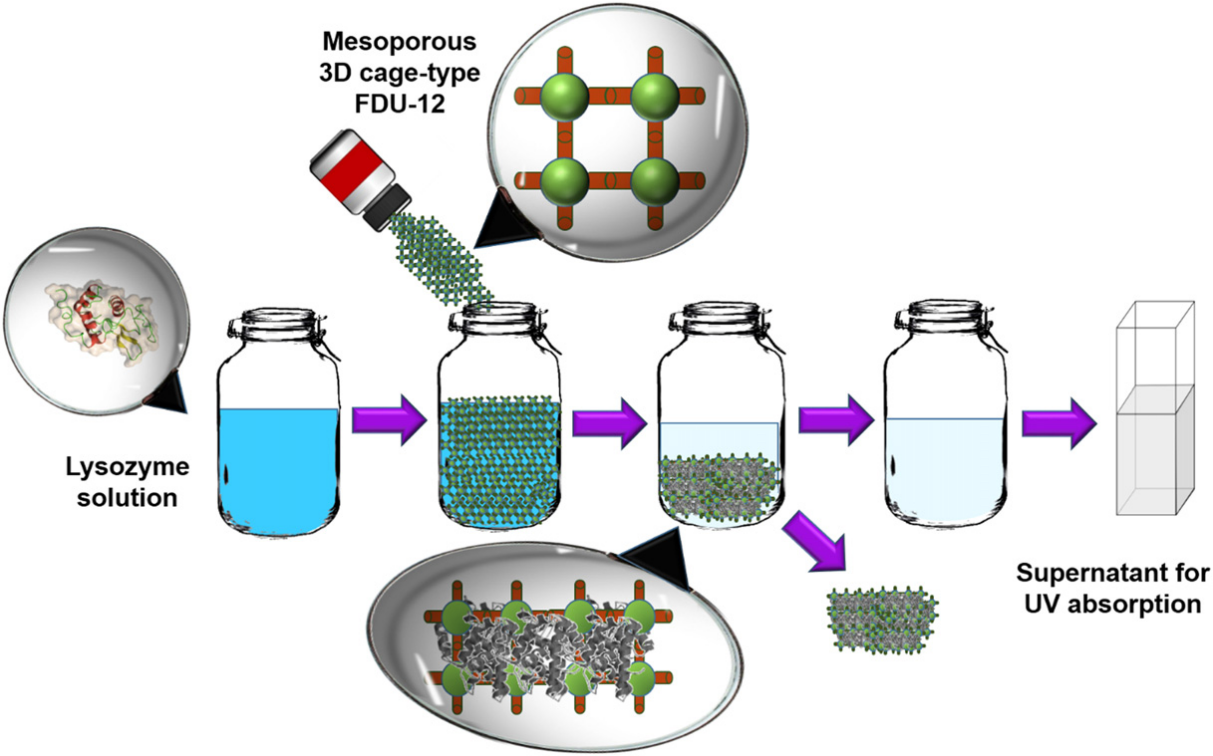
Schematic representation of the adsorption process using lysozyme on FDU-12 mesoporous silica.

**Figure 9.**
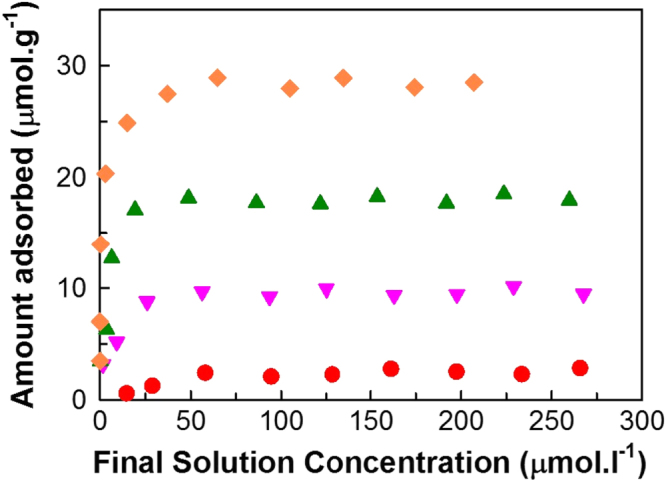
Adsorption of lysozyme over FDU-12-H prepared at different temperatures with an aging duration of 2 h (●) 100 °C (▼) 150 °C (▲) 180 °C and (♦) 200 °C.

The efficiency of FDU-12 synthesized with a short aging period was tested by performing adsorption studies using lysozyme. A schematic representation of the adsorption of lysozyme over FDU-12-HX samples is shown in figure 8. A standard optimized procedure [33] with a buffer concentration of 25 mM and a solution pH of 11 was used in the adsorption experiments. Figure 9 shows the adsorption isotherms of lysozyme adsorbed onto the FDU-12-H200 samples at the solution pH of 11 wherein the amount of lysozyme adsorbed per unit weight of the mesoporous adsorbent FDU-12 with different pore diameters is plotted against the equilibrium concentration of lysozyme solution. Optimized adsorption depends on the selection of pH which in this case is at pH 11 and is near the isoelectric point of lysozyme [30, 34]. At this pH, it is believed that the coulombic repulsive forces present in the adsorbate molecules are minimized, which significantly enhances the close packing of the protein molecules [26]. Hence, there is a maximum protein adsorption seen near the isoelectric point where the hydrophobic interaction is stronger than the electrostatic interaction, due to the neutral charge between the amido groups of lysozyme, siloxane bridges of FDU-12 and intermolecular originating from the amido groups on the lysozyme surface. The adsorption near the isoelectric point also supports the close packing of the protein molecules into the mesoporous cage by decreasing the solubility of proteins and thereby enhancing good hydration of carbonate ions under optimal ionic concentration of the buffer (25 mM). Figure 9 reveals that the lysozyme adsorption increases with an initial sharp escalation from a minimum of about 3 *μ*mol g^−1^ for FDU-12-H100 to a maximum of 29 *μ*mol g^−1^ for FDU-12-X200.

The rate and extent of adsorption is purely attributed to the textural parameters of the materials synthesized at different temperatures. It is found that the specific surface area, pore volume and pore diameters of the silica samples influence the amount of lysozyme adsorbed. More importantly, the size of the pore diameters, which spread over a broad range from 9.9 to 15.6 nm, is bound to have control over the amount of lysozyme molecules that can be immobilized into the 3D spherical pores of the FDU-12-HX materials. It can be clearly seen from figure 9 that the FDU-12-HX with the largest pore diameter and the highest pore volume offers the highest lysozyme adsorption capacity. The size of the pores not only has an influence over the diffusion of lysozyme into vacant sites in the cavities but also tends to permit close-fitting of the biomolecules possibly resulting in more layers of adsorption into the 3D cage-type mesoporous network. The 3D structure of FDU-12 with excellent textural parameters supports a high adsorption and superior mass transfer capacity of the adsorbate.

The adsorption capacity of FDU-12 prepared in this work was compared with other 1D and 3D materials. Table 4 gives a summary of the silica and carbon adsorbents used for the adsorption of lysozyme molecules. This comparison table gives brief information regarding the textural parameters, their aging duration and the amount of lysozyme adsorbed, which clearly shows the critical impact of the textural parameters and synthesis conditions on the amount of guest molecules adsorbed on the surface and into the porous channels of the 3D cage-type silica framework. It can be seen from table 4 that the adsorption capacity of FDU-12 is much higher than that of other samples studied, except KIT-6-150 which has the highest pore volume and 3D structure but requires a longer aging time of 24 h. These results revealed that the adsorbent with 3D cage-like network systems allow not only an easy access for lysozyme molecules to even the interior parts of the pores without any difficulties, but also easy diffusion via the pores that oriented in all different directions [33]. Thus, it can be concluded that a well-ordered 3D structure, large pore diameter and pore volume are essential for easy access to the adsorption sites thereby accommodating large amounts of biomolecules into mesoporous spherical cages.

**Table 4.** Comparison of the quantity of lysozyme adsorbed over adsorbents with different structures and textural parameters.

Adsorbent	Aging duration (h)	*A*_BET_ (m^2^ g^−1^)	*V*p (cm^3^ g^−1^)	*d*_p_ (nm)	Quantity of lysozyme adsorbed (*μ*mol g^−1^)
FDU-12-H200	2	387.0	0.94	15.64	29
FDU-12-M200 [31]	2	332.8	0.98	13.08	26
FDU-12-413 [13]	72	281	0.78	12.3	6.94
MCM-41 [35]	48	1036	0.83	2.7	1.94
PMO [36]	24	908.7	1.12	7.4	13.5
SBA-15 [36]	24	885	1.24	8.89	17.5
KIT-6-150 [33]	24	555	1.53	11.3	57.2
KIT-5-150 [37]	24	470	0.75	5.7	4.99
CMK-3-150 [33]	48	1350	1.6	6.5	22.9
CKT-3A-150 [34]	24	1600	2.1	5.2	26.5

From the variety of mesoporous carbon and silica species studied in the literature, it can also be seen that there is a long aging duration involved in the synthesis process of silica. In the case of mesoporous carbons, their synthesis involves the silica framework which has to be synthesized first and takes more than 5 days for the preparation of the samples including the dissolution of the silica template. There are even more materials with a high surface area and pore volume available in the literature, which also require a longer synthesis time. However, with such short aging durations (2 h) as shown in our report, and yet with such high pore volume and large pore diameters, we could achieve a comparatively high amount of lysozyme adsorption into the cage-type 3D mesoporous channels of the FDU-12-HX materials. Such improvements in expediting the aging treatment could speed up processes, culminating in ultra-fast synthesis at the industrial scale and prove to be a boon towards catalysis, adsorption, energy storage, drug delivery, separation processes and other biomedical applications.

## 4. Conclusions

We demonstrated the high temperature synthesis of FDU-12 with well-ordered structure and large pore diameter using a non-ionic surfactant. This simple strategy allowed us not only to significantly reduce the hydrothermal treatment time from 72 to 2 h, which is particularly important for practical applications, but also to prepare a material with tunable pore diameter and large pore volume. The XRD results confirmed that the well-ordered 3D mesoporous structure is still retained and the interconnected cage-type pores are formed even at 200 °C within the aging time of 2 h. The pore diameters could be tuned from 9.9 to 15.6 nm by changing the synthesis temperature from 100 to 200 °C. Among the samples studied, FDU-12-HX synthesized at 200 °C with 2 h aging displayed the highest pore volume of 0.93 cm^3^ g^−1^. We also demonstrated that the high temperature synthesis also supports the stability of the walls through perfect and quick silanol group condensation and further increases the hydrophobic nature of the sample. The prepared samples have been employed as adsorbents for the adsorption of lysozyme molecules. Among the samples studied, the sample with the largest pore diameter and the highest pore volume showed the highest adsorption capacity (29 *μ*mol g^−1^). We believe that this simple approach would definitely set a benchmark for much more efficient synthesis of FDU-12 and can be used for the synthesis of a variety of mesoporous materials with different structures and pore diameters within a short duration, which would create a platform for the easy commercialization of these materials and offer various application possibilities.
